# FACTORS ASSOCIATED TO QUALITY OF LIFE IN CHILDREN AND ADOLESCENTS WITH CYSTIC FIBROSIS

**DOI:** 10.1590/1984-0462/2020/38/2018397

**Published:** 2020-06-19

**Authors:** Nelbe Nesi Santana, Célia Regina Moutinho de Miranda Chaves, Christine Pereira Gonçalves, Saint Clair dos Santos Gomes

**Affiliations:** a Instituto Nacional de Saúde da Mulher, da Criança e do Adolescente Fernandes Figueira, Rio de Janeiro, RJ, Brasil.

**Keywords:** Cystic fibrosis, Quality of life, Chronic obstructive pulmonary disease, Pediatrics, Spirometry, Fibrose cística, Qualidade de vida, Doença pulmonar obstrutiva crônica, Pediatria, Espirometria

## Abstract

**Objective:**

To verify the association between quality of life, functional capacity and clinical and nutritional status in children and adolescents with cystic fibrosis (CF).

**Methods:**

Cross-sectional study, including patients from eight to 18 years old with CF. Quality of life, functional capacity, nutritional status and clinical status were evaluated with the Cystic Fibrosis Questionnaire; the 6-minute walk test (6MWT) and manual gripping force (MGF); the height percentiles for age and body mass index for age and respiratory function test, respectively. Pearson and Spearman correlation tests and logistic regression were used to analyze the data.

**Results:**

A total of 45 patients, 13.4±0.5 years old, 60% female, 60% colonized by *Pseudomonas aeruginosa* and 57.8% with at least one F508del mutation participated in the study. When assessing the perception of quality of life, the weight domain reached the lowest values, and the digestive domain, the highest. In the pulmonary function test, the forced expiratory volume of the first second was 77.3±3.3% and the 6MWT and MGF presented values within the normal range. There was an association between quality of life and functional capacity, nutritional status and clinical status of CF patients.

**Conclusions:**

The study participants had good clinical conditions and satisfactory values of functional capacity and quality of life. The findings reinforce that the assessment of quality of life may be important for clinical practice in the management of treatment.

## RESUMO

**Objetivo:**

Verificar associação entre qualidade de vida (QV), capacidade funcional e estados clínico e nutricional em crianças e adolescentes com fibrose cística (FC).

**Métodos:**

Estudo transversal incluindo pacientes de oito a 18 anos de idade com FC. A QV, a capacidade funcional, o estado nutricional e o estado clínico foram avaliados por meio do Questionário de Fibrose Cística; do teste de caminhada dos 6 minutos (TC6M) e da força de preensão manual (FPM); dos percentis de estatura para a idade e do índice de massa corporal (IMC) para a idade; e da prova de função respiratória, respectivamente. Para a análise dos dados, utilizaram-se os testes de correlação de Pearson e de Spearman e a regressão logística.

**Resultados:**

Participaram do estudo 45 pacientes com 13,4±0,5 anos, sendo 60% do sexo feminino, 60% colonizados por *Pseudomonas aeruginosa* e 57,8% apresentando pelo menos uma mutação F508del. Ao avaliar a percepção da QV, o domínio peso alcançou os escores mais baixos e o digestório, os mais altos. Na prova de função pulmonar, o volume expiratório forçado do primeiro segundo médio foi 77,3±3,3%, e o TC6M e a FPM apresentaram valores na faixa de normalidade. Observou-se associação da QV com a capacidade funcional, o estado nutricional e o estado clínico dos pacientes com FC.

**Conclusões:**

Os participantes do estudo apresentaram boas condições clínicas e valores satisfatórios de capacidade funcional e QV. Os achados reforçam que a avaliação da QV pode ser importante para a prática clínica, no manejo do tratamento.

## INTRODUCTION

Cystic fibrosis (CF) is a genetic, autosomal, recessive disease, more common in Caucasians, which manifests itself in many patients in the first years of life.^1^ ^,^ ^2^ The disease is characterized by a dysfunction of the cystic fibrosis transmenbrane conductance regulator (CFTR), responsible for regulating the transport of sodium, chlorine and water through epithelial membranes.^2^ ^,^ ^3^ The prevalence of CF varies around the world: from one in 1,400 inhabitants in Ireland to one in 3,500 in the United States.^4^ According to the 2016 Brazilian Cystic Fibrosis Registry, there are 4,654 individuals with CF in Brazil, with São Paulo, Minas Gerais, Rio Grande do Sul, Bahia and Rio de Janeiro States leading the list of the highest prevalences.^5^

Respiratory manifestations are responsible for 90% of morbidity and mortality rates and the multisystemic components of the disease, such as comorbidities of the respiratory, endocrine and digestive systems, and lead to important limitations, impacting the individual’s quality of life (QoL) and functional capacity.^6^ Some studies demonstrate that CF patients have reduced functional capacity in relation to healthy individuals.^7^ ^,^ ^8^ Functional capacity is understood by the individual’s ability to perform relevant activities and tasks of the daily routine, encompassing all body functions.^9^ Therefore, it is considered as an important indicator for assessing the QoL of patients with CF.

In addition to respiratory manifestations, nutritional impairment is essential for the prognosis of individuals with CF, since it is a predictor of survival and is directly associated to lung function and, consequently, to the morbidity and mortality of these patients.^10^ With the advancement of medicine and the emergence of new therapies over the last decades, the mean survival in CF has significantly increased, reaching 40 years in developed countries.^11^ Thus, the evaluation of the QoL of these patients is highly relevant, seeking their optimization.

The WHO defines Quality of Life as “an individual’s perception of their position in life in the context of the culture and value systems in which they live and in relation to their goals, expectations, standards and concerns.”^12^ Thus, for achieving a satisfactory QoL the individual needs to develop a good relationship in the aspects that permeate the social, psychological and physical domains, inserting their expectations in the context in which they live. This way, it is possible to achieve a healthy life, integrating physical and mental health.^13^

Knowledge of the QoL and functional capacity of the patient with CF, from childhood to adulthood, can help to infer the impact of the manifestations of the disease, as well as the numerous therapies in the daily routine, allowing them to be adjusted so that these individuals increase their survival with quality. Therefore, the aim of this study was to verify the association between QOL, functional capacity and clinical and nutritional status in children and adolescents with CF.

## METHOD

A cross-sectional study was carried out as a result of the first stage of evaluations of the cohort of CF patients followed by a reference center located in Rio de Janeiro State, which features a multidisciplinary team composed of doctors, physiotherapists, nutritionists, nurses, psychologists and social workers, which is responsible for the care of approximately 165 CF patients in quarterly consultations segmented by the type of bacterial colonization presented at the time of the consultation, that is, scheduled in groups according to the bacteria colonized in sputum.

All children and adolescents aged between eight and 18 years old and with a CF diagnosis confirmed by the presence of two mutations in the CFTR gene, as agreed by the Cystic Fibrosis Foundantion,^14^ who attended the scheduled multidisciplinary consultation were invited to participate in the study. The age range was defined according to the age range presented in the formulas for predicting the values obtained in the tests and with the ability to read and write to complete the questionnaires. Individuals with acute disease, requiring hospitalization up to 30 days before the tests, chronic oxygen-dependent hypoxemia or any condition that prevented the procedures from being performed were excluded from the study. Data were collected, and tests were carried out from July to December 2017, on the day of the follow-up consultation at the outpatient clinic, by a single researcher.

Variables obtained up to three months in advance and related to demographic characteristics (gender and age), clinical (pulmonary function test, bacterial colonization and type of genetic mutation) and nutritional (weight, height, body mass index - BMI, BMI percentile for age - BMI/A, and height percentile for age - H/A) were collected from medical records. In addition, functional capacity and QoL were assessed by the researcher.

The assessment of functional capacity was performed with the 6-minute walk test (6MWT), as recommended by the American Thoracic Society (ATS).^15^ The 6MWT is considered a submaximal capacity test and integrates the response of all systems involved during walking, including pulmonary, cardiovascular, neuromuscular, metabolic and psychosomatic systems.^15^ It is a safe test, easy to be performed, reproducible, validated and well tolerated, resulting in the one that best relates to the performance of the patient in the activities of daily living. The test assesses the maximum distance the patient can walk for six minutes in a 30 m flat corridor, defined every 3 m and bounded by two cones at its ends, in which the participant was instructed to walk as fast as possible. The test can be stopped at the patient’s request or when the saturation is less than 80%. In this study, no interruptions were observed during the test. To assess the distance covered, a prediction equation based on Brazilian children and adolescents was used, making it possible to adjust for some confounding variables such as gender, age, weight and height.^16^

In addition to the 6MWT, dynamometry was performed, which is considered a test to characterize the functional muscle status that assesses upper limb muscle strength (upper limbs). This test was applied as recommended by the American Society of Hand Therapists (ASHT) with the patient comfortably seated, positioned with the shoulder slightly adducted, elbow flexed at 90° and forearm and wrist in a neutral position. The patient was instructed to perform the maximum hand grip for 3 s with their dominant upper limb. The Jamar^®^ dynamometer (Rio de Janeiro, Brazil) was used, and the value considered was the average of the three measurements obtained.^17^ Just like with the 6MWT, a prediction equation was used to adjust the Hand Grip Strength (HGS) according to confounding variables, and the analyzed value was the percentage value of what was predicted according to the formula.^18^

To assess QoL, the Cystic Fibrosis Questionnaire (CFQ) was applied, self-completed for children or adolescents with a command of reading and writing. The guardian must not fill in for the child, according to the rules for applying the instrument. This questionnaire was developed by Quittner et al. and evaluates QoL in patients with CF from childhood to adulthood.^19^ In 2006, Rosov et al. translated and validated the CFQ into Portuguese.^20^ The questionnaire for patients has two versions: for children aged six to 11 years old, 12 and 13 years old and for teenagers/adults as from 14 years old; and for parents/guardians of children aged between six and 13 years old. The CFQ considers the physical, image, digestive, respiratory, emotional, social, food, treatment, vitality, health, social role and weight domains. Each domain has a score and its sum generates the total score, whose values can vary from zero to one hundred. The main advantages of this instrument are the use of the dimensions recommended by the WHO for the assessment of QoL and its easy application in clinical practice.

Patients’ pulmonary function impairment was assessed by the percentage of forced expiratory volume in the first second (FEV_1_), the forced vital capacity (FVC) and the FEV_1_/FVC ratio, achieved in relation to what was predicted, obtained from the pulmonary function test performed by professionals in the Function Test sector at the National Institute of Health for Women, Children and Adolescents Fernandes Figueira (IFF/Fiocruz) with Jaeger spirometer, MasterScope^®^ (VIASYS Healthcare, Hoechberg, Germany). The classification adopted was: normal (FEV_1_>80%), mild ventilation disorder (FEV_1_ between 79 and 70%), moderate ventilation disorder (FEV_1_ between 60 and 69%), moderately severe ventilation disorder (FEV_1_ between 50 and 59%), severe ventilatory disorder (FEV_1_ between 35 and 49%), and very severe ventilatory disorder (FEV_1_<35%). The technique for performing the exam and the reference values followed the recommendations of the ATS.^21^ The function tests performed within a period of up to six months of the pneumofunctional evaluation were considered valid for the study.

The categorical variables were described by their absolute and percentage frequencies; the numerical ones, by the mean and standard deviation. The strength of association of the variables considered with QoL was evaluated by the *Odds Ratio* (OR). The bivariate analysis provided the crude OR; the logistic regression provided the OR adjusted for the occurrence of the CFQ score under 80 with a 95% confidence interval (95% CI). The chi-square test was used to assess statistically significant differences for categorical variables. Spearman’s linear correlation analysis was used to identify variables related to the CFQ domains. A very weak correlation was considered when the correlation coefficient (R) was less than 0.19; weak when the (R) varied between 0.20 and 0.39; moderate with the (R) between 0.40 and 0.69; strong when the (R) varied between 0.7 and 0.89; and very strong with the (R) between 0.9 and 1.00.^22^ Student’s t-test was used for numerical variables, when the normality of the distribution was observed, or the ­Mann-Whitney test, when the normality of the distribution could not be identified. The Kolmogorov-Smirnov (KS) test was used to verify the normality of the data. All analyzes were performed using the Statistical Package for the Social Sciences (SPSS) version 23, with a significance level of 0.05.

The study was approved by the Research Ethics Committee (CEP) with Human Beings of IFF/Fiocruz, by CAAE 52272115.0.0000.5269 and by Opinion No. 2.133.819. All participants signed the informed consent form and their guardians signed the free and informed consent form.

## RESULTS

Of the 165 patients registered at the referral center, 76 met the inclusion criteria. Of these, 29 were excluded for cognitive impairment (4), chronic hypoxemia (2), need for hospitalization (1) and failure to attend appointments scheduled during the collection period (22). In addition, two teenagers refused to participate in the study. Thus, 45 patients with a mean age of 13.4 ± 0.5 years old were evaluated. Of the total, 60% were female and 60% were colonized by *Pseudomonas aeruginosa*. In the pulmonary function test, the mean FEV_1_ was 77.3 ± 3.3%, with 48.9% of the sample presenting mild ventilatory disorder and 4.4%, very severe ventilatory disorder. In addition, both the distance walked during the 6MWT and the HGS showed values close to normal (Table 1).

**Table 1:** Characterization of the sample and comparison of the variables studied according to the categorization of qualifty of life.

	Total (n=45)	Quality of life<80 (n=28)	Quality of life≥80 (n=17)	p-value
**Gender (male)**	40% (18)	**28.6% (8)**	**58.8% (10)**	0.040
Age (years old)	13.4±0.5	13.2±3.6	13.9±2.5	0.542
Bacterial colonization				
Negative	26.7% (12)	32.1% (9)	17.7% (3)	0.200
*Pseudomonas aeruginosa*	60% (27)	50.0% (14)	76.5% (13)	
Others	13.3% (6)	17.9% (5)	5.9% (1)	
**Genetic mutation**				
**F508del/F508del**	17.8% (8)	**28.6% (8)**	**0.0% (0)**	**0.020**
**F508del/other**	40% (18)	**28.6% (8)**	**58.8% (10)**	
**Other/other**	42.2% (19)	**42.9% (12)**	**41.2% (7)**	
H/A	25.8±20.0	23.1±16.4	30.3±24.7	0.400
BMI/A	33.8±24.1	34.1±22.9	33.5±26.7	0.791
FEV_1_ (%)	77.3±3.3	75.6±23.2	80.1±20.6	0.664
FEV_1_/FVC (%)	82.2±11.7	**79.9±12.6**	**86.1±8.9**	**0.040**
6MWT (%)	96.9±1.6	95.2±11.7	99.7±9.2	0.201
**HGS (%)**	74.8±2.2	**71.6±13.3**	**80.0±16.1**	**0.050**

H/A: height percentile for age; BMI/A: BMI percentile for age; FEV_1_: percentage of forced expiratory volume in the first second; FVC: forced vital capacity; 6MWT: 6-minute walk test; HGS: Hand Grip Strength. Highlighted, the data with statistically significant difference.

As for the assessment of QoL, according to the CFQ, the average total score was 75.7 ± 11.4. The mean scores in relation to the domains varied between 66.7 ± 30.6 for weight, and between 87.2 ± 24.5 for digestion (Table 2).

**Table 2:** Correlation between the total score and the Cystic Fibrosis Questionnaire domains and the functional, nutritional and clinical variables.

CFQ domains	Medium values	6MWT (%)	HGS (%)	H/A	BMI/A	FEV_1_ (%)	FVC (%)	FEV_1_/FVC (%)
**Physical**	74.4±19.8	0.14	0.20	0.04	0.02	0.13	0.08	**0.30**
**Social role**	85.2±21.9	0.15	0.32	-0.53	-0.004	0.36	0.27	**0.48**
**Vitality**	77.5±13.3	0.04	0.12	-0.31	**-0.50**	0.06	-0.02	0.23
**Emotional**	75.3±17.9	0.003	0.15	-0.07	**-0.31**	-0.22	-0.20	-0.05
**Social**	72.4±17.5	-0.08	0.15	-0.15	-0.09	-0.24	**-0.30**	0.04
Body	69.4±30.1	0.12	0.13	-0.18	0.005	-0.16	-0.11	-0.04
Food	82.2±23.5	0.13	0.06	0.04	0.02	-0.04	-0.16	0.20
**Treatment**	75.6±18.9	**0.39**	0.19	-0.11	0.16	**0.33**	0.22	0.24
**Health**	75.5±21.5	0.32	0.19	-0.41	-0.06	0.33	0.17	**0.50**
**Weight**	66.7±30.6	0.21	-0.03	-0.39	**0.49**	0.02	-0.11	0.09
**Respiratory**	73.8±15.8	-0.06	**0.30**	0.07	-0.14	0.16	0.04	**0.44**
**Digestion**	87.2±24.5	-0.06	0.10	0.13	0.00	0.16	0.08	**0.34**
**Total**	75.7±11.4	0.19	**0.29**	-0.57	-0.02	0.05	-0.41	**0.33**

CFQ: Cystic Fibrosis Questionnaire; 6MWT: 6-minute walk test; HGS: Hand Grip Strength; H/A: height percentile for age; BMI/A: BMI percentile for age; FEV_1_: percentage of forced expiratory volume in the first second; FVC: forced vital capacity. The values in bold represent statistically significant correlations, with p≤0.05.

The correlation analysis between the CFQ domains, the distance covered in the 6MWT and the HGS shows that there is a significant correlation only between the hedged distance and the treatment domain, and between the HGS and the respiratory domain and the total score (Table 2).

Regarding the studied nutritional variables, there was a significant correlation from weak to moderate, but negative, between the BMI/A and the domains related to vitality and emotion, and a positive correlation between the BMI/A and the weight domain. The level of the association varied between -0.3 (emotional domain) and -0.5 (vitality domain). There was no correlation between H/A and the CFQ domains (Table 2).

Regarding the association between the variables of the respiratory function test and the CFQ domains, in general, the level of correlation varied from weak to moderate. FEV_1_% was associated to the treatment domain, whereas FVC% was associated to the social domain. The FEV_1_/FVC relationship was associated to the physical, social role, health, respiratory and digestive domains and the total score, and the function test variable showed the best correlation with the CFQ domains, with the value of the coefficient of correlation ranging from 0.3 (total score) to 0.5 (health) (Table 2).

When categorizing the total QoL score between less than 80 and greater than or equal to 80, the variables gender, type of genetic mutation, FEV_1_/FVC and HGS ratio showed differences between categories (Table 1). In addition, when performing logistic regression based on these categories, it was found that only the FEV_1_/FVC ratio and HGS were associated to QoL in patients with CF (Table 3).

**Table 3:** Final adjustment of the logistic regression model for the occurrence of a Cystic Fibrosis Questionnaire under 80.

Variables	B	p-value	OR	CI95%
**HGS (%)**	**0.56**	0.04*	**1.06**	**1.00-1.12**
FEV_1_ (%)	-0.46	0.11	0.96	0.90-1.01
**FEV_1_/FVc (%)**	**0.12**	0.03*	**1.12**	**1.01-1.25**

B: intercept of logistic regression; OR: *Odds Ratio*; 95% CI: 95% confidence interval; HGS: Hand Grip Strength; FEV_1_: forced expiratory volume in the first second; FVC: forced vital capacity; *p≤0.05. The initial model was adjusted for the variables gender, age, bacterial colonization, height, body mass index and the distance covered in the 6-minute walk test. The final model presented as statistically significant variables the handgrip strength and the ratio between the forced expiratory volume in the first second and the forced vital capacity.

## DISCUSSION

In the present study, the sample evaluated showed clinical, functional capacity and QoL values close to normal, despite the fact that most individuals are adolescents. Given the chronic and progressive nature of the disease, this result is extremely important and corroborates the worldwide trend of increasing life expectancy for this population affected by CF.^11^

Although the sample had good clinical conditions and acceptable QoL levels, 60% of the individuals were colonized by *Pseudomonas aeruginosa*. It is known that colonization by *Pseudomonas aeruginosa* increases the number of medications in the daily routine and accelerates the decline in lung function.^23^ However, in the present study, it was not associated to the decline in QoL.

The male gender, when compared to the female gender, was associated to better QoL values, which is corroborated by the literature that shows a lower risk of death.^24^ Therefore, the male gender seems to have less disease severity and better function pulmonary disease, which leads to better functional capacity and increased survival. Arrington-Sanders et al. obtained similar results when evaluating 98 American patients with CF, aged between 10 and 18 years old.^25^ In our study, the male gender showed higher scores in most domains of the questionnaire in relation to the female gender, associating less impact on QoL in boys.^26^

In addition, in the present study, individuals were stable at the time of assessment, which contributed to the observation of adequate values, given that the exacerbations and hospitalizations can affect both the functional capacity and the perception of QoL.^26^ ^,^ ^27^

Although the weight domain reached the lowest score in relation to the assessment of QoL, the digestive domain reached the highest values. This discrepancy can be explained by the difference between the questions about these domains, in which the weight domain assesses the difficulty in gaining weight, and the digestive, issues such as abdominal pain, diarrhea, etc.^28^ Therefore, it can be concluded that, in this sample, the digestive symptoms are less impactful for QoL than the difficulty in gaining weight. Borawska-Kowalczyk et al., when evaluating 70 Polish adolescents aged from 14 to 18, also found a higher score in the digestive domain, corroborating the low impact of symptoms related to this domain on QoL of patients with CF.^29^

The domain of CFQ treatment correlated with the distance covered in the 6MWT and FEV_1_, demonstrating the importance of its performance for the assessment of the functional capacity and clinical status of children and adolescents with CF. Donadio et al. showed in their study that the functional capacity assessed by the 6MWT in children and adolescents with CF is a predictor of hospitalization risk.^30^ Therefore, it can be inferred that the adequate treatment is inversely associated to the risk of hospitalization.

Of the variables studied, the FEV_1_/FVC ratio was the one with the highest number of associations with the QoL domains, correlating with the physical, role in society, health, respiratory and digestive domains, and even with the total QFQ score. Therefore, the analysis of this relation is important for the studied population, since most studies consider only FEV_1_. Some studies show an association between the total QoL score and the respiratory function test.^26^ ^,^ ^31^ In line with the findings, Dill et al., when studying American adults with CF, concluded that the total score was associated to FEV_1_,^25^ which was also found in a multicenter-national epidemiological study with CF patients in the United States.^31^

The assessment of QoL and functional capacity of patients with chronic respiratory diseases is an important routine for both the patient and the multidisciplinary team. The need for long and complex treatments compromises the physical, mental and social well-being of children and adolescents with this chronic disease.^20^ The findings demonstrate the impact and benefits of treatment on these individuals, facilitating clinical decision and optimizing survival with quality.^32^

The CFQ has been widely used as a self-reported assessment measure in clinical practice, allowing the health team to access the benefits of treatment and its contribution or impact on the QoL of each patient. Thus, when identifying the factors in the questionnaire that have the greatest impact on the QoL of patients with CF, professionals can prioritize and/or optimize these factors, so that the individual can survive with QoL.^32^ ^,^ ^33^ ^,^ ^34^

One point that deserves to be highlighted was the role of children and adolescents as the main informants of research in obtaining data related to their own QoL, because they are those who daily experience the extensive care routine, generating more reliable information. Thus, knowledge of these data can assist the multidisciplinary team in conducting the treatment properly.

As study limitations, there is the small sample size due to the rareness of the disease, preventing the generalization of findings. In addition, the transversal cut does not allow inference of causality. One further limitation is the absence of data as to pancreatic insufficiency, which can influence the variables evaluated and the acute conditions, excluded in the present study, and does not allow the generalization of results.

From this study, we can conclude that the children and adolescents in the sample had good clinical conditions and satisfactory values, both related to functional capacity and QoL. When assessing QoL with the CFQ, the weight domain was the least scored, whereas the digestive domain reached the highest score. The findings reinforce that the assessment of QoL, in addition to being simple to perform, can be important for clinical practice in the management of treatment, because it assesses its repercussion among other factors in the daily routine of CF patients.
